# Complete Genome Sequence of a *bla*_KPC-2_-Positive Klebsiella pneumoniae Strain Isolated from the Effluent of an Urban Sewage Treatment Plant in Japan

**DOI:** 10.1128/mSphere.00314-18

**Published:** 2018-09-19

**Authors:** Tsuyoshi Sekizuka, Koji Yatsu, Yuba Inamine, Takaya Segawa, Miho Nishio, Norimi Kishi, Makoto Kuroda

**Affiliations:** a Pathogen Genomics Center, National Institute of Infectious Diseases, Toyama, Tokyo, Japan; U.S. Centers for Disease Control and Prevention

**Keywords:** *Klebsiella pneumoniae*, *bla*_KPC-2_, carbapenemase, effluent, urban sewage

## Abstract

Antimicrobial resistance genes (ARGs) and the bacteria that harbor them are widely distributed in the environment, especially in surface water, sewage treatment plant effluent, soil, and animal waste. In this study, we isolated a KPC-2-producing Klebsiella pneumoniae strain (GSU10-3) from a sampling site in Tokyo Bay, Japan, near a wastewater treatment plant (WWTP) and determined its complete genome sequence. Strain GSU10-3 is resistant to most β-lactam antibiotics and other antimicrobial agents (quinolones and aminoglycosides). This strain is classified as sequence type 11 (ST11), and a core genome phylogenetic analysis indicated that strain GSU10-3 is closely related to KPC-2-positive Chinese clinical isolates from 2011 to 2017 and is clearly distinct from strains isolated from the European Union (EU), United States, and other Asian countries. Strain GSU10-3 harbors four plasmids, including a *bla*_KPC-2_-positive plasmid, pGSU10-3-3 (66.2 kb), which is smaller than other *bla*_KPC-2_-positive plasmids and notably carries dual replicons (IncFII [pHN7A8] and IncN). Such downsizing and the presence of dual replicons may promote its maintenance and stable replication, contributing to its broad host range with low fitness costs. A second plasmid, pGSU10-3-1 (159.0 kb), an IncA/C2 replicon, carries a class 1 integron (containing *intI1*, *dfrA12*, *aadA2*, *qacEΔ1*, and *sul1*) with a high degree of similarity to a broad-host-range plasmid present in the family *Enterobacteriaceae*. The plasmid pGSU10-3-2 (134.8 kb), an IncFII(K) replicon, carries the IS*26*-mediated ARGs [*aac*(*6'*)*Ib-cr* , *bla*_OXA-1_, *catB4* (truncated), and *aac*(3)*-IId*], *tet*(A), and a copper/arsenate resistance locus. GSU10-3 is the first nonclinical KPC-2-producing environmental *Enterobacteriaceae* isolate from Japan for which the whole genome has been sequenced.

**IMPORTANCE** We isolated and determined the complete genome sequence of a KPC-2-producing K. pneumoniae strain from a sampling site in Tokyo Bay, Japan, near a wastewater treatment plant (WWTP). In Japan, the KPC type has been very rarely detected, while IMP is the most predominant type of carbapenemase in clinical carbapenemase-producing *Enterobacteriaceae* (CPE) isolates. Although laboratory testing thus far suggested that Japan may be virtually free of KPC-producing *Enterobacteriaceae*, we have detected it from effluent from a WWTP. Antimicrobial resistance (AMR) monitoring of WWTP effluent may contribute to the early detection of future AMR bacterial dissemination in clinical settings and communities; indeed, it will help illuminate the whole picture in which environmental contamination through WWTP effluent plays a part.

## Abstract (precis)

We isolated and determined the complete genome sequence of a KPC-2-producing K. pneumoniae strain from a sampling site in Tokyo Bay, Japan, near a wastewater treatment plant (WWTP). In Japan, the KPC type has been very rarely detected, while IMP is the most predominant type of carbapenemase in clinical carbapenemase-producing *Enterobacteriaceae* (CPE) isolates. Although laboratory testing thus far suggested that Japan may be virtually free of KPC-producing *Enterobacteriaceae*, we have detected it from effluent from a WWTP. Antimicrobial resistance (AMR) monitoring of WWTP effluent may contribute to the early detection of future AMR bacterial dissemination in clinical settings and communities; indeed, it will help illuminate the whole picture in which environmental contamination through WWTP effluent plays a part.

## INTRODUCTION

Antimicrobial resistance (AMR) is a global health crisis linked to increased and often unrestricted antibiotic use in the clinical and veterinary fields. WHO guidelines have been published for the infection prevention and control (IPC) of carbapenem-resistant *Enterobacteriaceae* (CRE), carbapenem-resistant Acinetobacter baumannii (CRAB), and carbapenem-resistant Pseudomonas aeruginosa (CRPsA) in health care facilities (1). These bacteria have the potential to facilitate the widespread transmission of AMR via mobile genetic elements through the processes of natural competence, transformation, and plasmid transconjugation that can occur in any environment. For these reasons, it has been concluded that the early recognition of CRE-CRAB-CRPsA should be a high priority to allow evidence-based recommendations to be provided. Furthermore, specific and required IPC practices and procedures should be conducted to effectively prevent the occurrence of these infections and control their spread in acute health care facilities.

In addition to clinical settings, AMR genes (ARGs) and AMR bacteria are widely distributed in the environment, particularly in surface waters (2), sewage treatment plant effluents (3), and soils and animal wastes (4). No direct evidence has yet been uncovered for the transmission of ARGs to humans through the environment, and the extent to which environmental factors contribute to human exposure should be quantified and compared to clinical and veterinary data. A systematic literature review was recently conducted on human exposure to AMR bacteria, including extended-spectrum β-lactamase (ESBL)-producing *Enterobacteriaceae* in the environment (5). The results indicated that AMR bacteria can be detected at exposure-relevant sites, including recreational areas, and in drinking water, ambient air, shellfish, and fresh produce.

In particular, the widespread detection of carbapenemase-producing *Enterobacteriaceae* (CPE) in the environment is an emerging environmental issue with potentially serious public health implications. Notably, Xu et al. reported the isolation of Klebsiella pneumoniae carbapenemase (KPC)-producing *Citrobacter* and *Aeromonas* isolates from sampling sites near a wastewater treatment plant (WWTP) by the Shifeng River in China (6). An article reviewing antibiotic resistance in China highlighted the issues concerning the enrichment and dissemination of ARGs in the environment in China (7), as well as the need to mitigate the spread of AMR in the environment, particularly under the “One-Health” perspective.

In this study, we isolated and determined the complete genome sequence of a KPC-2-producing K. pneumoniae strain from a sampling site in Tokyo Bay, Japan, near a wastewater treatment plant (WWTP). The first Japanese KPC-2-producing K. pneumoniae clinical isolate, K. pneumoniae strain Kp3018, was classified as sequence type 11 (ST11) and was isolated from a patient treated at a Brazilian hospital in 2012 (8). Since then, there have been no reports documenting additional KPC-2-producing K. pneumoniae in Japan, indicating that the Japanese environment may be almost free of KPC-producing K. pneumoniae. This is the first report on the genomic features of a nonclinical KPC-2-producing *Enterobacteriaceae* isolate from Japan.

## RESULTS

### CRE isolate from WWTP effluent.

A large, mucoid colony on CHROMagar ESBL plate was selected for whole-genome sequence (WGS) analysis and was identified as K. pneumoniae and designated GSU10-3. The GSU10-3 isolate carries *bla*_KPC-2_, exhibited resistance to meropenem, and had a positive reaction for the Carba NP test. Further antimicrobial susceptibility testing showed that K. pneumoniae GSU10-3 is resistant to most β-lactam antibiotics with Etest (Table 1) and to other antimicrobial agents, including trimethoprim-sulfamethoxazole, ciprofloxacin, kanamycin, gentamicin, streptomycin, minocycline, and tetracycline as determined by disk diffusion test. The string test showed that it had negative stretch and was a low-viscosity colony.

**TABLE 1:** Etest results on the K. pneumoniae GSU10-3 isolate

Antibiotic(s)	MIC (µg/ml)
Meropenem	>8
Meropenem-EDTA	>2
Imipenem	>32
Cefotaxime	>256
Cefotaxime-clavulanic acid^*a*^	>1
Aztreonam	>256
Sulbactam-ampicillin	>256

a Clavulanic acid present at a fixed concentration of 4 µg/ml.

### Whole-genome sequence analysis of K. pneumoniae GSU10-3.

Basic information regarding the complete chromosome and plasmid sequences for K. pneumoniae GSU10-3 is shown in Table 2. An analysis of the complete chromosomal DNA sequence classifies GSU10-3 as ST11 (based on the results of multilocus sequence typing [MLST] analysis). The strain possesses plasmids harboring multiple AMR genes (Table 2). The *wzi* gene sequence corresponds to the gene in capsular genotype K47 in a homology search against *wzi* sequence database (9). Strain GSU10-3 does not show a hypermucoviscosity phenotype by the string test, and regulator of mucoid phenotype A (*rmpA*) (10) and transcriptional activator (*rmpA2*) (10) genes were not found in both the chromosome and those plasmids.

**TABLE 2:** Whole-genome information for K. pneumoniae GSU10-3 from WWTP effluent

Replicon	% GC	Length (bp)	Inc type	Drug resistance gene(s)	GenBank ID
Chromosome	57.4	5,478,620		*bla*_SHV-1_, *fosA6*	AP018671
pGSU10-3-1	51.6	159,072	IncA/C2	*dfrA12*, *aadA2*, *qacEdelta1*, *sul1*, *qnrA1*, *aac*(*3*)*-IId*, *blaTEM-1B*, *aph*(*6*)*-Id*, *aph*(*3''*)*-Ib*, *sul2*	AP018672
pGSU10-3-2	52.8	134,879	IncFII	*tet*(*A*), *aac*(*6'*)*Ib-cr*, *bla*_OXA-1_, *catB4*, *aac*(*3*)*-IIa*	AP018673
pGSU10-3-3	54.2	66,250	IncN and IncFII (pHN7A8)	*bla*_KPC-2_, *fosA3*	AP018674
pGSU10-3-4	55.1	10,060	ColRNAI	Not found	AP018675

### Core genome phylogenetic analysis of K. pneumoniae GSU10-3.

**FIG 1:**
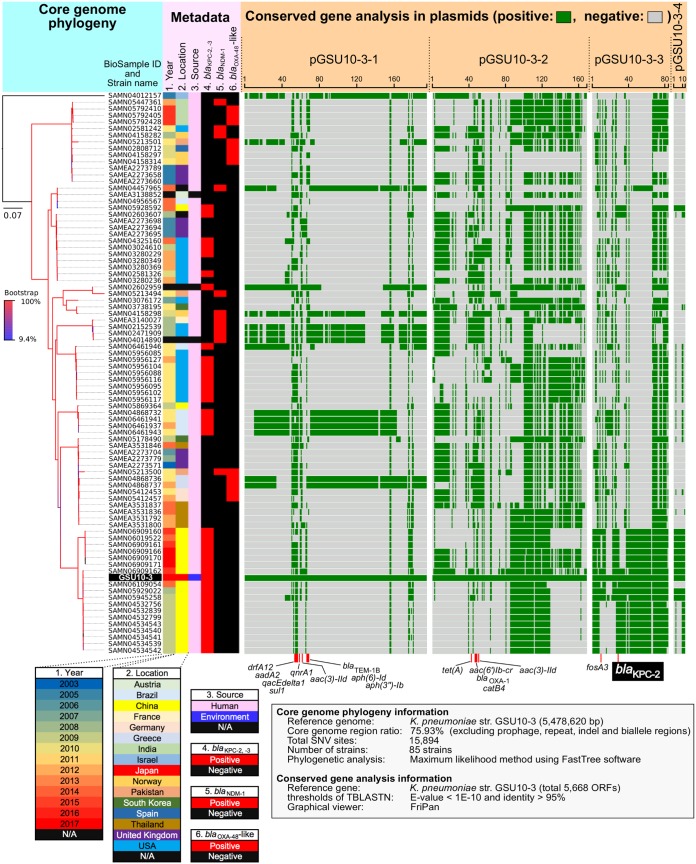
Representation of a core genome phylogeny and conserved gene analysis of K. pneumoniae strains. A complete picture of the core genome phylogeny of 85 strains of ST11 K. pneumoniae, including GSU10-3, is shown. The core genome phylogeny was constructed by the maximum likelihood method using the core genome region of strain GSU10-3 (75.93% of the genome; 4,159,904/5,478,620 bp), excluding repeat and recombination regions. The isolate year, location, source, and the specific β-lactamases are indicated by the different colors. Conserved genes (≥95% homology) shared with GSU10-3 as a reference genome are highlighted for the chromosome and four plasmids. Antimicrobial resistance genes are indicated below. Information for all tested strains is shown in Table S1 in the supplemental material. N/A, not available; str., strain.

To trace the potential sources of the GSU10-3 strain, we performed core genome phylogenetic analysis using 84 publicly available ST11 K. pneumoniae genome sequences (on 18 April 2018), including draft genomes (see the strain list in Table S1 in the supplemental material). To date, no environmental, ST11 K. pneumoniae genome sequence has been deposited in a public database. Thus, GSU10-3 is the first fully sequenced environmental isolate for which the genome sequence has been characterized. The core genome of those tested K. pneumoniae strains constitutes 75.93% of the genome (4,159,904/5,478,620 bp), and the phylogenetic analysis using 15,894 single-nucleotide variations (SNVs) indicated that strain GSU10-3 shares a common lineage with 19 KPC-2-positive Chinese isolates from human clinical specimens obtained from 2011 to 2017 and is clearly distinct from strains from the European Union (EU) (20 strains), United States (19 strains), and other Asian countries (14 strains) (Fig. 1).

10.1128/mSphere.00314-18.2 TABLE S1 Information on the Klebsiella pneumoniae strains used in whole-genome SNV phylogenetic analysis (Fig. 1). Download Table S1, PDF file, 0.03 MB. Copyright © 2018 Sekizuka et al. 2018 Sekizuka et al. This content is distributed under the terms of the Creative Commons Attribution 4.0 International license.

### Structural comparison of KPC-2 plasmids.

**FIG 2:**
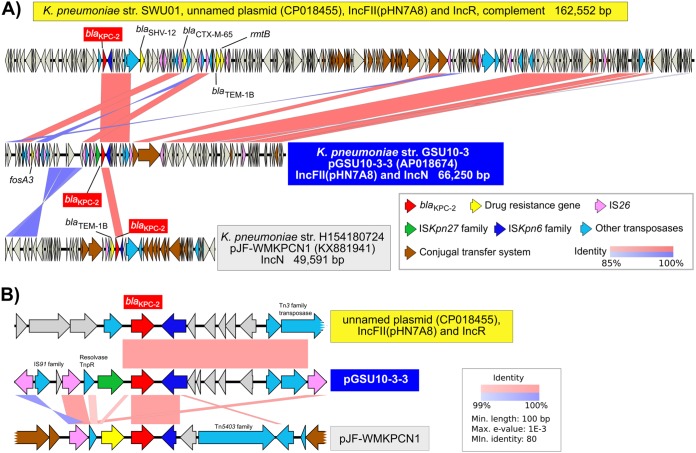
Structural comparison of the *bla*_KPC-2_-positive plasmids. (A) K. pneumoniae SWU01 (BioSample SAMN06109054) was isolated from a clinical blood specimen from a human in China in 2015, K. pneumoniae H154180724 (GenBank KX881941) was isolated from a human in the United Kingdom. (B) Comparison of gene structure around *bla*_KPC-2_ is highlighted from panel A. The *bla*_KPC-2_ gene in pGSU10-3-3 is flanked by IS*Kpn27* and IS*Kpn6*. Min., minimum; Max., maximum.

A conserved gene analysis of the four GSU10-3 plasmids indicated that the KPC-2 encoding plasmid (pGSU10-3-3 in strain GSU10-3) shares a number of conserved genes with the above-mentioned 19 KPC-2-positive Chinese isolates (Fig. 1), while the other three plasmids show partial homology to other K. pneumoniae strains. For instance, pGSU10-3-1 (IncA/C replicon plasmid) is similar to plasmids of K. pneumoniae strains isolated in Brazil (four isolates), France (one isolate), Germany (two isolates) and the USA (two isolates). The KPC-2-containing plasmid pGSU10-3-3 (66.2 kb) is on a relatively small IncFII (pHN7A8) replicon compared with other KPC-2-containing plasmids (see Fig. S1), and a pairwise alignment shows that some of the genes involved in the conjugal transfer system (dark brown open reading frames [ORFs] in Fig. 2A) have been removed in pGSU10-3-3. Multiple IS*26* might contribute to the loss of conjugation potential by the excision with a possible homologous recombination event. In addition, pGSU10-3-3 has an additional IncN replicon (inverted alignment with dark blue in Fig. 2A) compared to the IncN plasmid pJF-WMKPCN1. Structural comparisons suggested that pGSU10-3-3 carries two Inc replicons (IncFII [pHN7A8] and IncN) and appears to have partially lost conjugational machinery.

Comparison of genes around *bla*_KPC-2_ suggested that multiple insertion sequnces (ISs) could be involved in the acquisition of *bla*_KPC-2_, resulting in the gene structure IS*26–tnpR*–IS*Kpn27–bla*_KPC-2_–IS*Kpn6* (Fig. 2B). Part of the gene structure (IS*Kpn27–bla*_KPC-2_–IS*Kpn6*) is similar to that of a KPC-2-positive IncP-6 plasmid (p121SC21-KPC2, GenBank accession no. or identifier [ID] LT992437) in Citrobacter freundii CF121SC21 isolate obtained from wastewater in 2012 (11), while eight copies of IS*26* insertions (pink ORFs in Fig. 2A and B) are a notable genetic feature of pGSU10-3-3 in this study. IS*26* is one of the ubiquitous ISs in the family *Enterobacteriaceae*. IS*26* sequences are deposited as follows: 5,176 sequence entries in *Klebsiella,* 4,255 in Escherichia coli, 1,764 in *Acinetobacter*, 1,508 in *Salmonella*, 980 in *Enterobacter*, and 1,605 in other members of the *Enterobacteriaceae* family were identified by BLASTp homology search (17 July 2018), suggesting that IS*26* could be one of the marked ISs in *Klebsiella* species.

### ARGs.

In addition to *bla*_KPC-2_ described above, ARGs are listed in Table 2, and the ARG position in each plasmid is indicated in the linear replicon representation in Fig. 1 and Fig. S1 in the supplemental material.

10.1128/mSphere.00314-18.1 FIG S1 A whole-plasmid nucleotide sequence search of GSU10-3 plasmids pGSU10-3-1 (A), pGSU10-3-1 (B), pGSU10-3-1 (C), and pGSU10-3-1 (D) against complete plasmid sequence database (13,286 entries as of 5 May 2018). Schematic pairwise alignment of the plasmid is displayed with each significant homologous plasmid as a subject sequence. A red box and a coverage value indicate a region similarity at ≥80% nucleotide identity and shared regions (as a percentage), respectively. Download FIG S1, PPT file, 2.3 MB. Copyright © 2018 Sekizuka et al. 2018 Sekizuka et al. This content is distributed under the terms of the Creative Commons Attribution 4.0 International license.

The pGSU10-3-1 plasmid (159,072 bp) is an IncA/C2 replicon that carries a class 1 integron (*intI1*, *dfrA12*, *aadA2*, *qacEΔ1*, and *sul1*), *qnrA1*, *aac*(*3*)*-IId*, *bla*_TEM-1B_, *aph*(*6*)*-Id*, *aph*(*3''*)*-Ib*, *sul2*, and multiple ISs (including an IS*91* family member, three IS*26* elements, and members of the IS*Ehe3*, IS*903D*, and IS*1182* families). A whole-plasmid search indicates that pGSU10-3-1 shares similarity to the broad-host-range plasmids present in K. pneumoniae and other members of the *Enterobacteriaceae*, including *Aeromonas*, *Citrobacter*, *Enterobacter*, *Escherichia*, *Proteus,* and *Vibrio* (Fig. S1).

The pGSU10-3-2 plasmid (134,879 bp) is an IncFII(K) replicon that carries IS*26*-mediated ARGs [*aac*(*6'*)*Ib-cr*, *bla*_OXA-1_, *catB4* (truncated), and *aac*(3)*-IId*], *tet*(A), and a copper/arsenate resistance locus. The copper resistance proteins encoded by the *cop* operon mediate the sequestration of copper in the periplasm (12), and the ArsAB membrane complex functions in arsenic resistance as an anion-translocating ATPase (13). Such metal resistance genes are likely to increase the persistence, fitness, and propagation of the plasmid in the bacterial host under the conditions of environmental stress present in sewage. A whole-plasmid search indicated that pGSU10-3-2 shares similarity to the only K. pneumoniae-related plasmid, which has a narrow host range.

As described above, the pGSU10-3-3 plasmid (66,250 bp) carries *bla*_KPC-2_ and *fosA3*.

The pGSU10-3-4 plasmid (10,060 bp) is a ColRNAI replicon, and ARGs or virulence determinants have not been found on it.

## DISCUSSION

In this study, we isolated a KPC-2-producing K. pneumoniae isolate (GSU10-3) from the effluent of a WWTP in Tokyo Bay, Japan. WWTPs could be a primary source of most AMR bacteria and ARGs in aquatic environments, probably due to the partial purification that occurs during the wastewater treatment processes. A recent study of a Japanese WWTP identified GES- and imipenemase (IMP)-, but not KPC-type, CPE isolates from wastewater (14).

In Japan, IMP is the most predominant type of carbapenemase in clinical CPE isolates (15–17). VIM, OXA48, GES, and NDM carbapenemases are more rarely detected, while the KPC type is very rarely detected, suggesting that Japan may be virtually free of KPC-producing *Enterobacteriaceae*. In contrast, KPC is predominant in isolates from other East Asian countries. Twenty-four million travelers from Asian countries visited Japan in 2017, with more than a 20% annual rate of increase (as reported by the Japan National Tourism Organization), indicating that in addition to specific, local types of CPE, comprehensive testing should be conducted for every type of imported CPE.

Isolate GSU10-3 is classified as ST11 K. pneumoniae, which is closely related to the dominant clone of KPC-producing K. pneumoniae in China (18). For instance, the KPC-producing K. pneumoniae isolates from central China between 2009 and 2014 were clonally related, with ST11 being the reservoir for the *bla*_KPC-2_ and ESBL genes (*bla*_CTX-M-55_). These findings demonstrated the high prevalence of carbapenemase- and ESBL-producing K. pneumoniae in central China (19). An outbreak of KPC-2-producing K. pneumoniae (ST11) in a neonatal ward has been reported (20). Hypervirulent and multidrug-resistant K. pneumoniae strains pose a significant threat to public health, and a recent study reported a hospital outbreak in China that involved the dissemination of ST11 with KPC-2-producing K. pneumoniae (21). GSU10-3 in this study showed low-mucoviscosity colony formation and does not carry hypermucoviscosity-related regulator genes (*rmpA* and *rmpA2*), suggesting that GSU10-3 might not be the markedly hypervirulent isolate, instead of multiple antimicrobial resistance.

The GSU10-3 isolate possesses the KPC-2-positive plasmid pGSU10-3-3, which contains the Inc replicons IncF and IncN in the same relaxase MOB_F_ family (22). It has been reported that the IncN group can colocalize as a fusion with IncF plasmids (23), suggesting that although pGSU10-3-3 is smaller than other KPC-2-related plasmids, such downsizing could contribute to its stable replication with dual replicons. This scenario would contribute to a broad host range, leading to a rather low fitness cost compared to that of larger KPC-2 plasmids observed in clinical isolates (24) (see Fig. S1 in the supplemental material).

When KPC-producing K. pneumoniae causes an epidemic of carbapenem-resistant *Enterobacteriaceae* (CRE) in health care settings in developed countries, the spread primarily occurs through patient-to-patient transmission (25). So-called superspreaders are individuals who are likely to have high rectal CRE concentrations, and such CRE carriers are responsible for partial shedding and may play a central role in CRE transmission (26). There have been many reports of CRE in hospital water, and the majority of these reports have described associated clinical outbreaks in intensive care settings, affecting the critically ill and immunocompromised. Drains, sinks, and faucets are the most frequently colonized by CRE, and the most appropriate disinfection method remains unclear. However, it is likely that the replacement of colonized water reservoirs may be required for long-term clearance (27).

Previous studies have sought to establish a possible role for the natural environment in the transmission of clinically relevant AMR bacteria to humans. However, quantitative data analysis from exposure-relevant sites and environmental compartments were not sufficient to determine the abundance of AMR bacteria that pose a significant risk for human exposure, although AMR bacteria have been detected in diverse environments, including wastewater, as shown in this study. The increase in selective pressures due to the overuse of extended-spectrum cephalosporins (widely available as generics) has contributed to the global dissemination of CTX-M-type ESBL strains in all types of environments. Healthy carriers of CTX-M-type ESBL-harboring bacteria are another major public health concern, because carriage rates are increasing, particularly in South East Asia and Eastern Mediterranean regions. Carriers from these regions have the potential to spread these bacteria to other communities (28). Indeed, surfers appear to be at risk of exposure to and colonization by clinically important AMR E. coli in coastal waters (29). Additionally, short-term international travel to Vietnam increases the carriage risk of colistin-resistant, ESBL-producing E. coli (30). Raw vegetables and local foods appear to become contaminated with AMR bacteria due to insufficient hygiene in irrigation water systems, making this a potential means by which such bacteria disseminate to humans via retail vegetables (31).

In conclusion, AMR monitoring of WWTP effluent may contribute to the detection of on-going AMR bacterial dissemination in clinical settings and communities. A comprehensive approach will be required to uncover the bigger picture in which environmental contamination through WWTP effluent plays a part.

## MATERIALS AND METHODS

### Bacterial isolation.

The upper effluent flow of an urban wastewater treatment plant (WWTP) was collected on 23 August 2017 (N35.654861, E139.833358) in Tokyo Bay, Japan. Five hundred milliliters of the effluent was filtered through a polyethersulfone (PES) filter membrane with a pore size of 0.22 µm (Vacuum Filtration "rapid"-Filtermax; TPP Techno Plastic Products, Trasadingen, Switzerland). A quarter of the membrane was incubated with 20 ml of LB broth supplemented with 1 µg/ml meropenem at 37°C for 14 h to select for bacteria with reduced carbapenem susceptibility. The culture (1 to 10 µl) was spread on the CHROMagar ESBL plates (CHROMagar, Paris, France) and incubated at 37°C for 18 h. A colony with a unique morphology, designated K. pneumoniae GSU10-3, was isolated for further molecular genomic analysis. Hypermucoviscosity phenotype K. pneumoniae was checked by the string test (32).

### Antimicrobial susceptibility and CPE screening tests.

Antimicrobial susceptibility testing was performed using Etest (bioMérieux, Marcy-l’Étoile, France) and disk diffusion methods under CLSI M100-S28 (33). Carbapenemase production was assessed using a Carba NP test, as described previously (34).

### Whole-genome sequence (WGS) analysis.

Genomic DNA from the isolated strain was purified by collecting cells from a 5-ml overnight culture grown in tryptic soy broth (TSB). The cell pellet was resuspended in 500 µl of TE10 (10 mM Tris [pH 8.0] and 10 mM EDTA) supplemented with 500 µl phenol-chloroform, and the cells were subsequently lysed by bead beating for 10 min in ZR BashingBead lysis tubes (Zymo Research, Irvine, CA, USA) attached to a vortex adapter (MO BIO Laboratories, Qiagen, Carlsbad, CA, USA). After centrifugation at 10,000 rpm for 5 min, the upper phase was further purified using a Qiagen DNA purification kit (Qiagen, Germany). Short DNA fragments (approximately 0.5 kb) for paired-end sequencing were generated using an Illumina XT DNA library kit (Illumina). Whole-genome sequencing of the paired-end library was performed using an Illumina NextSeq 500 platform with a 300-cycle NextSeq 500 reagent kit v2 (150-mer paired ends; median coverage, ×96).

The complete genome sequence of the strain was determined using a PacBio Sequel sequencer for long-read sequencing (Sequel SMRT Cell 1M  v2 [four/tray]; Sequel sequencing kit v2.1; insert size, approximately 10 kb). Purified genomic DNA (∼200 ng) was used to prepare a SMRTbell library using a SMRTbell template prep kit 1.0 (PacBio, Menlo Park, CA, USA) with barcoding adapters according to the manufacturer’s instructions. Sequencing data were produced with more than 100-fold coverage and assembled using the assembly program SMRT Link v5.

A *de novo* assembly was performed using Canu version 1.4 (35), minimap version 0.2-r124 (36), racon version 1.1.0 (37), and Circlator version 1.5.3 (38). Error correction of tentative complete circular sequences was performed using Pilon version 1.18 with Illumina short reads (39). Annotation was performed in Prokka version 1.11 (40), InterPro v49.0 (41), and NCBI-BLASTP/BLASTX.

Circular representations of complete genomic sequences were visualized using GView server (42). Antimicrobial resistance (AMR) genes were identified by homology searching against the ResFinder database (43).

### Comparative genome sequence analysis.

All publicly available draft genome sequences of K. pneumoniae strains were retrieved (at least ×40 read coverage; see Table S1 in the supplemental material) and compared using bwaMEM read mapping against the K. pneumoniae GSU10-3 complete genome sequence (AP018671) as a reference. After excluding repeat regions and six prophage sequences from the whole-genome sequence, 75.9% of the genome was assigned as the core genome sequence among 85 collected strains, resulting in the identification of 15,894 single-nucleotide variations (SNVs) (Fig. 1). The core genome multilocus sequence typing (cgMLST) was performed using the SNVs described above, and the phylogeny was generated using the maximum likelihood phylogenetic method with FastTree v2.1.10.

Conserved gene sequence analysis among plasmids was performed with BLASTn searches (≥95% nucleotide [nt] identity), followed by visualization using FriPan (http://drpowell.github.io/FriPan/).

Comparative plasmid sequence analysis was performed with BLASTn searches (≥85% nt identity), followed by visualization using Easyfig (44).

### Accession number(s).

The complete, annotated genomic sequence of K. pneumoniae GSU10-3 was deposited in a public database (accession numbers AP018671, AP018672, AP018673, AP018674, and AP018675). The short- and long-read sequences for transcriptome sequencing (DNA-Seq) were deposited in the DNA Data Bank of Japan (BioProject PRJDB6962, BioSample SAMD00116246, DRA accession no. DRA006779).
