# Supplementary material for: Complete Genome Sequence of a blaKPC-2-Positive Klebsiella pneumoniae Strain Isolated from the Effluent of an Urban Sewage Treatment Plant in Japan
Source: mSphere. 2018 Sep 19;3(5):e00314-18. doi: 10.1128/mSphere.00314-18 (PMC6147131; doi:10.1128/mSphere.00314-18)
Supplement: TABLE S1 [file sph005182640st1.pdf]

Table S1. Information of *Klebsiella pneumoniae* strains used in whole genome SNV phylogenetic analysis (Fig. 1).

BioSample	strain	collection date	geographic location	host	isolation source
SAMN04012157	B86	2005	Israel	Homo sapiens	blood
SAMN02581242	MGH 69	2013	USA	Homo sapiens	Sputum
SAMN04158282	K66-45	2010	Norway	Homo sapiens	urine
SAMN05213501	CFSAN044571	2013	Pakistan	Homo sapiens	other body fluid
SAMN05447361	B20143	2014	India	Homo sapiens	blood
SAMN05792410	PM1842	2016	India	Homo sapiens	urine
SAMN05792428	PM138	2016	India	Homo sapiens	bronchoalveolar lavage
SAMN05792405	PM565	2016	India	Homo sapiens	blood
SAMEA2273658	k1205	2005	United Kingdom	Homo sapiens	blood
SAMEA2273660	k1209	2005	United Kingdom	Homo sapiens	blood
SAMEA2273789	k1206	2005	United Kingdom	Homo sapiens	blood
SAMN02808712	KP_ST11_OXA48	2012	Spain	Homo sapiens	blood
SAMN04158297	50625602	2012	Norway	Homo sapiens	urine
SAMN04158314	50806829	2013	Norway	Homo sapiens	blood
SAMEA3138852	IS33	N/A	Austria	N/A	N/A
SAMN04956567	KPC45	2015	Brazil	Homo sapiens	anal swab
SAMN04457965	KP38731	2015	N/A	Homo sapiens	N/A
SAMN05928592	XL-1	2015	China	Homo sapiens	blood
SAMN02603607	N/A	2010	N/A	Homo sapiens	72-year-old male with cerebral infarction after colon carcinoma resection
SAMEA2273698	k1485	2006	United Kingdom	Homo sapiens	blood
SAMEA2273694	k1446	2006	United Kingdom	Homo sapiens	blood
SAMEA2273695	k1449	2006	United Kingdom	Homo sapiens	blood
SAMN04325160	CHS241	2015	USA	Homo sapiens	Tissue
SAMN03024610	CAV1392	2011	USA	Homo sapiens	Sputum
SAMN03280229	CHS86	2014	USA	Homo sapiens	N/A
SAMN03280349	CHS208	2014	USA	Homo sapiens	N/A
SAMN03280236	CHS93	2014	USA	Homo sapiens	N/A
SAMN02581326	CHS 24	2012	USA	Homo sapiens	blood
SAMN03280369	CHS229	2014	USA	Homo sapiens	N/A
SAMN02602959	HS11286	N/A	N/A	N/A	N/A
SAMN05213494	CFSAN044563	2013	Pakistan	Homo sapiens	blood
SAMN03076172	101731	2008	USA	Homo sapiens	wound
SAMN03738195	YMC 2013/7/B3993	2013	South Korea	Homo sapiens	blood
SAMN04158298	50627996	2012	Norway	Homo sapiens	urine
SAMEA3140027	KPM_nasey	2010	France	Homo sapiens	N/A
SAMN02471909	ATCC BAA-2146	2010	USA	Homo sapiens	urine
SAMN02152539	ATCC BAA-2146	2010	USA	Homo sapiens	N/A
SAMN04014890	N/A	N/A	N/A	N/A	N/A
SAMN06461946	1853	2013	Brazil	Homo sapiens	catheter
SAMN05956085	1075_1947	2013	USA	Homo sapiens	urine
SAMN05956104	1287_2164	2013	USA	Homo sapiens	respiratory
SAMN05956088	1146_2028	2013	USA	Homo sapiens	urine
SAMN05956116	1430_2335	2013	USA	Homo sapiens	urine
SAMN05956127	1531_2471	2014	USA	Homo sapiens	respiratory
SAMN05956095	1194_2067	2013	USA	Homo sapiens	urine
SAMN05956117	1437_2342	2013	USA	Homo sapiens	urine
SAMN05956102	1280_2158	2013	USA	Homo sapiens	urine
SAMN05869364	kp10	2011	China	Homo sapiens	blood
SAMN04868732	12_BR_13	2013	Brazil	Homo sapiens	urine
SAMN06461943	1799	2013	Brazil	Homo sapiens	blood
SAMN06461937	4182	2014	Brazil	Homo sapiens	blood
SAMN06461941	1787	2013	Brazil	Homo sapiens	blood
SAMN05178490	YMC2011/11/B7578	2011	South Korea	Homo sapiens	blood
SAMEA2273571	k557	2003	United Kingdom	Homo sapiens	blood
SAMEA3531846	PB450	2015	Thailand	Homo sapiens	urine
SAMEA2273704	k1534	2007	United Kingdom	Homo sapiens	blood
SAMEA2273779	k2037	2009	United Kingdom	Homo sapiens	blood
SAMN05213500	CFSAN044570	2013	Pakistan	Homo sapiens	blood
SAMN05412453	Kp_Goe_822917	2013	Germany	Homo sapiens	skin swab

SAMN05412457	Kp_Goe_821588	2014	Germany	Homo sapiens	anal swab
SAMN04868736	16_GR_13	2013	Greece	Homo sapiens	Stool
SAMN04868737	17_GR_14	2014	Greece	Homo sapiens	Stool
SAMEA3531837	PB366	2015	Thailand	Homo sapiens	urine
SAMEA3531800	PB122	2014	Thailand	Homo sapiens	Peritoneal dialysis fluid (PDF)
SAMEA3531792	PB65	2014	Thailand	Homo sapiens	sputum
SAMEA3531836	PB327	2015	Thailand	Homo sapiens	sputum
SAMN06909160	WCHKP2	2016	China	Homo sapiens	N/A
SAMN06909170	WCHKP108	2017	China	Homo sapiens	N/A
SAMN06909171	WCHKP113	2017	China	Homo sapiens	N/A
SAMN06909161	WCHKP3	2016	China	Homo sapiens	N/A
SAMN06019522	WCHKP095649	2015	China	Homo sapiens	Secreta
SAMN06909166	WCHKP91	2017	China	Homo sapiens	N/A
SAMN06909162	WCHKP10	2016	China	Homo sapiens	N/A
SAMN06109054	SWU01	2015	China	Homo sapiens	blood specimen
SAMN05929022	KP6	2010	China	Homo sapiens	central venous catheter
**SAMD00116246**	**GSU-10-3**	**2017**	**Japan**	**Environment**	**sewage effluent**
SAMN05945258	KP41	2011	China	Homo sapiens	urine
SAMN04534543	TR262	2011	China	Homo sapiens	sputum
SAMN04532799	TR191	2011	China	Homo sapiens	abdominal drainage
SAMN04534539	TR198	2011	China	Homo sapiens	sputum
SAMN04534542	TRqt-49	2011	China	Homo sapiens	nose
SAMN04534541	TR200	2011	China	Homo sapiens	urine
SAMN04534540	TRqt-41	2011	China	Homo sapiens	nose
SAMN04532756	TR187	2011	China	Homo sapiens	sputum
SAMN04532839	TRqt-37	2011	China	Homo sapiens	nose
