# Supplementary material for: Complete Genome Sequence of a blaKPC-2-Positive Klebsiella pneumoniae Strain Isolated from the Effluent of an Urban Sewage Treatment Plant in Japan
Source: mSphere. 2018 Sep 19;3(5):e00314-18. doi: 10.1128/mSphere.00314-18 (PMC6147131; doi:10.1128/mSphere.00314-18)
Supplement: FIG S1 [file sph005182640sf1.ppt]

## Slide 1
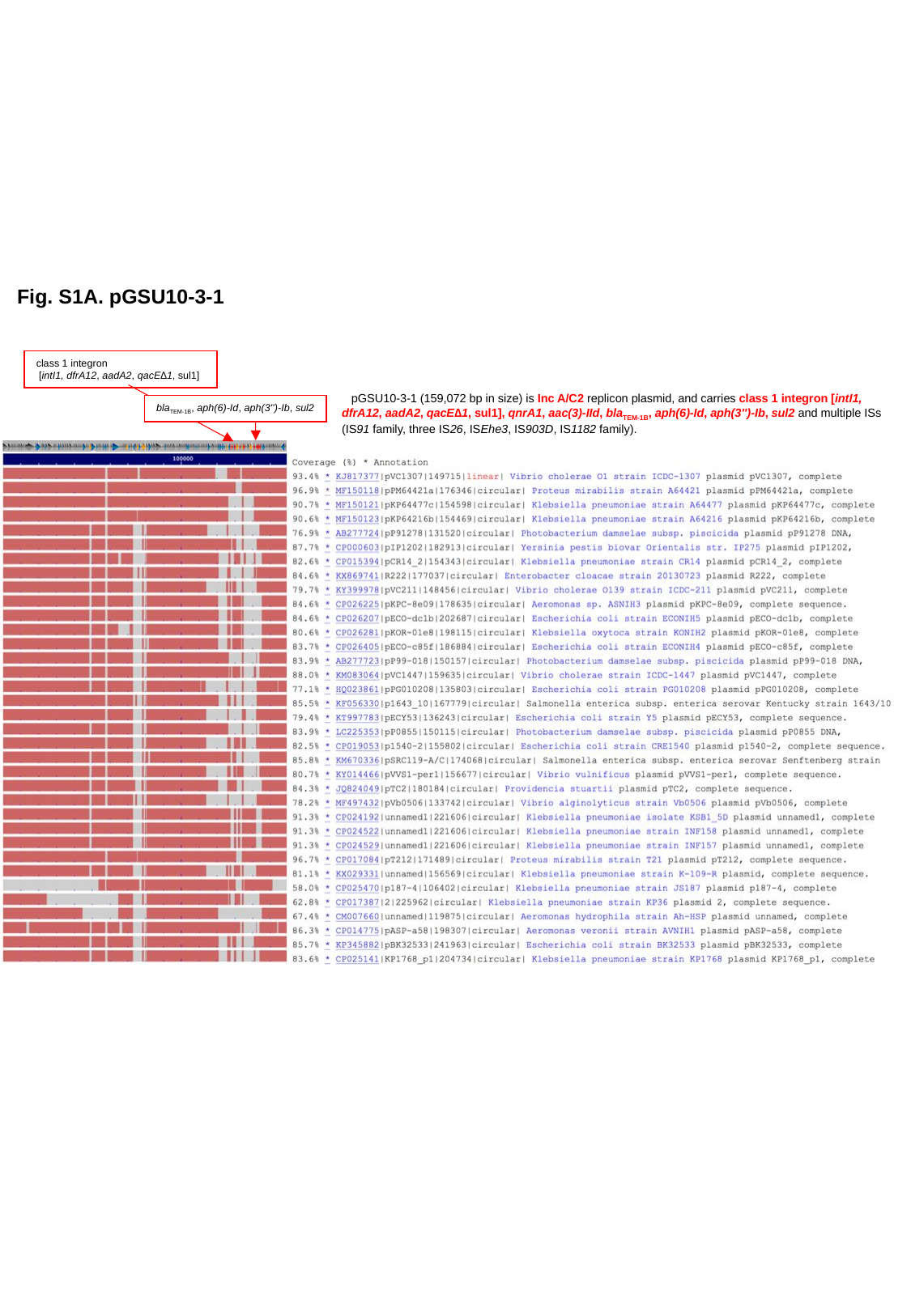

Fig. S1A. pGSU10-3-1
class 1 integron
 [intI1, dfrA12, aadA2, qacE∆1, sul1]
 pGSU10-3-1 (159,072 bp in size) is Inc A/C2 replicon plasmid, and carries class 1 integron [intI1, dfrA12, aadA2, qacE∆1, sul1], qnrA1, aac(3)-IId, blaTEM-1B, aph(6)-Id, aph(3'')-Ib, sul2 and multiple ISs (IS91 family, three IS26, ISEhe3, IS903D, IS1182 family).
blaTEM-1B, aph(6)-Id, aph(3'')-Ib, sul2

## Slide 2
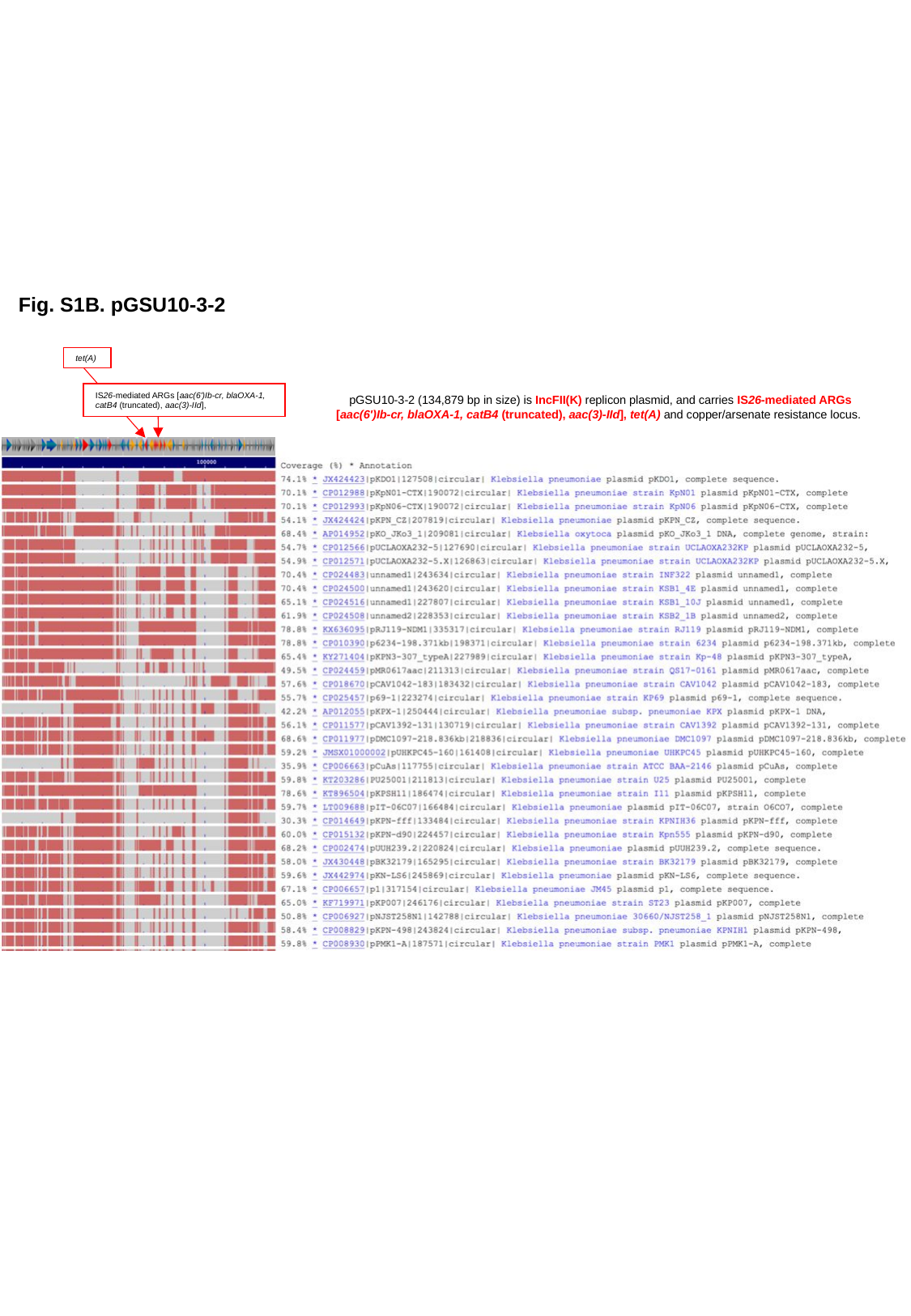

Fig. S1B. pGSU10-3-2
tet(A)
IS26-mediated ARGs [aac(6')Ib-cr, blaOXA-1, catB4 (truncated), aac(3)-IId],
 pGSU10-3-2 (134,879 bp in size) is IncFII(K) replicon plasmid, and carries IS26-mediated ARGs [aac(6')Ib-cr, blaOXA-1, catB4 (truncated), aac(3)-IId], tet(A) and copper/arsenate resistance locus.

## Slide 3
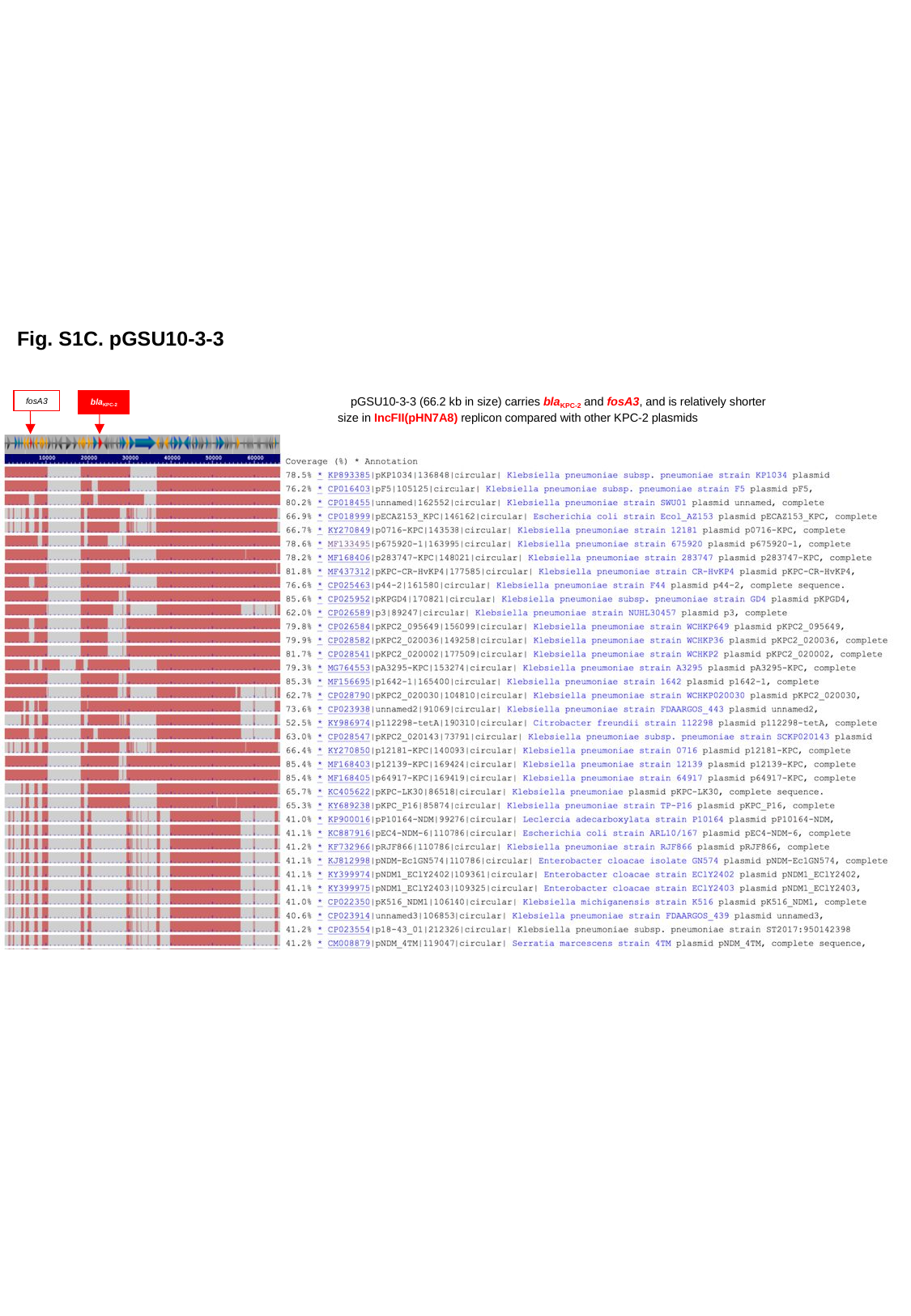

Fig. S1C. pGSU10-3-3
 pGSU10-3-3 (66.2 kb in size) carries blaKPC-2 and fosA3, and is relatively shorter size in IncFII(pHN7A8) replicon compared with other KPC-2 plasmids
fosA3
blaKPC-2

## Slide 4
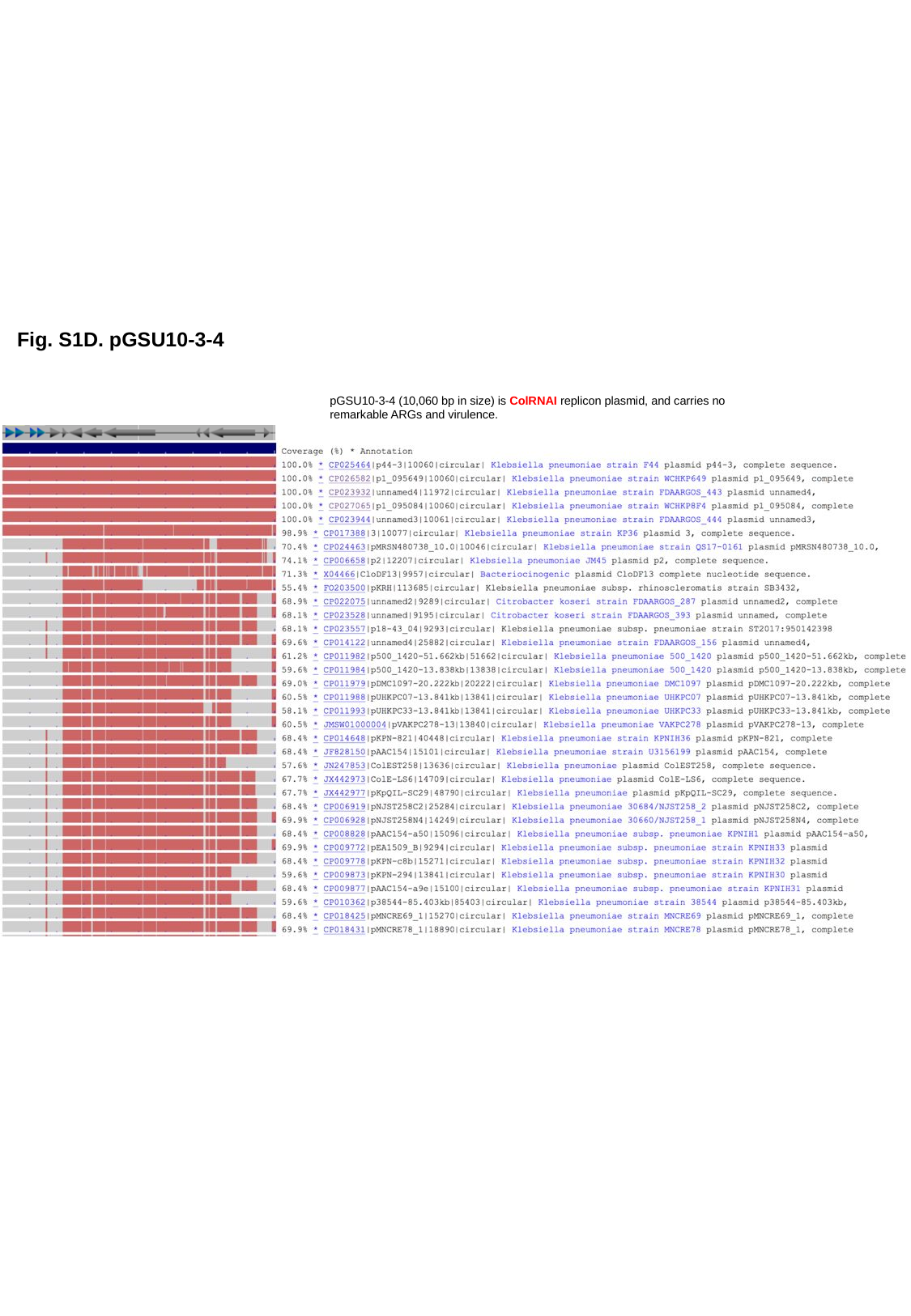

Fig. S1D. pGSU10-3-4
pGSU10-3-4 (10,060 bp in size) is ColRNAI replicon plasmid, and carries no remarkable ARGs and virulence.
